# Efficacy of Bacillus clausii UBBC-07 and Bacillus coagulans Unique IS-2 in Mitigating Pulmonary Parameters in Patients With Moderate COVID-19 Symptoms

**DOI:** 10.7759/cureus.76436

**Published:** 2024-12-26

**Authors:** Mehdi Ali Mirza, Rajiv Kumar Bandaru, Ushasree T

**Affiliations:** 1 Department of Pharmacology, Employees' State Insurance Corporation (ESIC) Medical College and Hospital, Hyderabad, IND; 2 Department of General Medicine, Employees' State Insurance Corporation (ESIC) Medical College and Hospital, Hyderabad, IND

**Keywords:** adjuvant therapy, bacillus clausii, bacillus coagulans, covid-19, d-dimer, gut-lung axis, probiotics, serum ferritin

## Abstract

Probiotics have shown efficacy in preventing and reducing infections caused by common viruses, including rotavirus, norovirus, hepatitis, human papillomavirus (HPV), human immunodeficiency virus (HIV), and herpes simplex virus (HSV). A randomized, double-blind, placebo-controlled, three-arm parallel-group study was conducted on 56 patients with moderate COVID-19 symptoms. Patients were randomly assigned to one of the three groups: standard treatment combined with *Bacillus clausii *UBBC-07, standard treatment combined with *Bacillus coagulans *Unique IS-2, or standard treatment with a placebo. The probiotics were administered as an oral suspension containing 2 billion spores, taken twice daily for 14 days. The placebo group received distilled water administered twice daily for 14 days. Clinical recovery and respiratory parameters were assessed, with C-reactive protein (CRP), serum ferritin, and lactate dehydrogenase (LDH) levels measured on days one, seven, and 14, while D-dimer and interleukin-6 levels were evaluated on days one and 14. There was a significant reduction (p < 0.01) in serum ferritin levels for both *Bacillus clausii* UBBC-07 and *Bacillus coagulans* Unique IS-2 compared to the placebo group. The median reduction in serum ferritin levels on day seven was 345 μg/L (39.2%) in the *Bacillus clausii* UBBC-07 group, 350 μg/L (40.7%) in the *Bacillus coagulans* Unique IS-2 group, and 270 μg/L (30.9%) in the placebo group. By day 14, the reductions were 665 μg/L (75.6%) for the *Bacillus clausii* UBBC-07 group, 630 μg/L (73.3%) for the *Bacillus coagulans* Unique IS-2 group, and 595 μg/L (69.1%) for the placebo group. D-dimer levels were significantly reduced by the end of treatment (day 14) in the *Bacillus coagulans* Unique IS-2 group compared to the placebo group, with median decreases of 0.36 μg/ml (60.0%) for *Bacillus coagulans* Unique IS-2, 0.21 μg/ml (36.8%) for *Bacillus clausii* UBBC-07, and 0.22 μg/ml (34.4%) for the placebo group. No significant differences were observed in other biomarkers, including CRP, LDH, and interleukin-6. No adverse effects related to probiotic administration were observed, and the intervention was well tolerated by all patients. In conclusion, *Bacillus coagulans* Unique IS-2 and *Bacillus clausii* UBBC-07 may be considered adjunct therapies for mitigating COVID-19 infections.

## Introduction

Coronavirus disease 2019 (COVID-19) is a recent pandemic disease caused by the severe acute respiratory syndrome coronavirus 2 (SARS-CoV-2). Its symptoms include fever, dry cough, shortness of breath, and upper and/or lower respiratory tract symptoms. It can also lead to fatigue, myalgia or headache, diarrhea, nausea, or a combination of these a few days after exposure [1,2].

Some of the risk factors for severe COVID-19 reactions include underlying chronic diseases, compromised immune status, advanced age, and a disturbed gut microbiota (dysbiosis).

Many studies have indicated that in COVID-19 patients, beneficial bacteria like lactobacilli and bifidobacteria are decreased with an increase in opportunistic pathogens such as *Clostridium ramosum* and *Clostridium hathewayi*. Decreases in levels of butyrate-producing bacteria* Faecalibacterium prausnitzii* and *Eubacterium* have also been observed [3-5]. Gut dysbiosis has been correlated with uncontrolled dysregulation of the host’s immune function leading to severe inflammatory reactions, i.e., an increased cytokine storm and disease severity, suggesting an association between a disturbed intestinal microbiota composition and severity of COVID-19 [6]. Various studies have described significant changes in the innate and adaptive immune systems in COVID-19 patients [7,8]. One of the main causes of this pro-inflammatory state is the reduction in commensal bacteria and their metabolites and hence it is suggested that supplementation with probiotics, which are beneficial bacteria, will reduce the severe inflammatory response in COVID-19. Moreover, probiotics have earlier demonstrated efficacy in the prevention and reduction of infections caused by common viruses such as rotavirus, norovirus, viruses that cause hepatitis, human papillomavirus, human immunodeficiency virus, and herpes simplex virus. Many studies have also reported the efficacy of probiotics in the treatment of respiratory viral infections, including respiratory syncytial virus and influenza A virus [9]. It is therefore believed that probiotics can help relieve COVID-19 symptoms by boosting immune host response and improving gut microbiota [10]. Several clinical trials have demonstrated the efficacy of probiotics as an adjunct to standard COVID-19 treatments. The probiotic strains used in these studies included *Lactobacillus acidophilus*,* Bifidobacterium infantis*, *Lactiplantibacillus plantarum*, *Pediococcus acidilactici*, *Lactococcus lactis*, *Lactobacillus rhamnosus*, *Bifidobacterium bifidum*, *Bifidobacterium longum* subsp. *infantis*, *Lactobacillus* *reuteri*, and* Streptococcus salivarius* [11].

It is suggested that probiotics act by multifactorial mechanisms to enhance antiviral immunity in the host. Some of the mechanisms are through inhibition of pathogen adherence, enhancement of epithelial barrier, and boosting of the immune system [12]. Probiotics can modulate cytokines, enhance IgA secretion, enhance phagocytosis, modulate the function of regulatory cells, induce dendritic cell maturation, strengthen the mucosal barrier, and minimize viral entry [13].

Most of the trials to evaluate the efficacy of probiotic strains on COVID-19 have included the *Lactobacillus* and *Bifidobacterium* species with hardly any studies on the *Bacillus* species, many of which have a long history of use. There have been a few studies with *Bacillus coagulans* in viral infections wherein consumption of *Bacillus coagulans* for 28 days increased CD3+CD69+ cells and interferon gamma levels after ex vivo exposure to a strain of adenovirus (AdenoVI) or influenza A (H3N2 Texas strain) [14].

The advantages of *Bacillus* over *Lactobacillus* and *Bifidobacterium* species are that they have a long shelf life and are stable at room temperature, eliminating the need for refrigeration during storage and transport. They survive the transit through the gastrointestinal tract and are resilient to most manufacturing processes. Moreover, most of them have a long history of use and are considered safe.

In this study, the efficacy of two well-known commercially available probiotic strains,* Bacillus coagulans* Unique IS-2 and *Bacillus clausii* UBBC-07, was evaluated in patients with mild to moderate symptoms of COVID-19. Mild cases were defined as those without shortness of breath or hypoxia, with normal oxygen saturation levels.

*Bacillus clausii* UBBC-07 and *Bacillus coagulans* Unique IS-2 are shelf-stable, safe, and well-documented probiotic strains [15,16]. Apart from playing a major role in maintaining digestive health [17-19], *B. clausii *UBBC-07 and *B. coagulans* Unique IS-2 have been found to possess anti-inflammatory and immunomodulatory properties [20]. In addition, *B. clausii* UBBC-07 has been found to be efficacious in reducing upper respiratory tract infections in children [21]. In vitro studies have highlighted the potential of *Bacillus clausii* against SARS-CoV-2 spike proteins [22].

In the present study, the impact of administering *Bacillus coagulans* Unique IS-2 and *Bacillus clausii *UBBC-07 on specific biomarkers associated with COVID-19 infection and inflammation was evaluated. The biomarkers assessed were C-reactive protein (CRP), which was assessed using a qualitative test, lactate dehydrogenase (LDH), serum ferritin, D-dimer, and interleukin-6 (IL-6).

Elevated levels of these markers are known to correlate with increased disease severity in COVID-19 patients. By monitoring these biomarkers, the study aimed to assess the potential of the probiotic strains as an adjunct in slowing the progression of the disease and influencing the inflammatory response.

CRP is an acute-phase protein produced by the liver in response to inflammation and is used to monitor disease progression and therapeutic response. Elevated CRP levels are commonly observed in COVID-19 patients. Serum ferritin is a well-known inflammatory marker and is linked with increased disease severity and mortality in COVID-19 patients [23]. LDH is an enzyme involved in the conversion of lactate to pyruvate during cellular respiration. Elevated LDH levels in COVID-19 patients are indicative of tissue damage and cellular injury, reflecting the severity of the disease [24]. Monitoring LDH levels provides insight into the extent of organ damage and overall prognosis. D-dimer is a fibrin degradation product that is elevated in conditions of coagulation and fibrinolysis. COVID-19 is associated with a high incidence of thrombotic events, and elevated D-dimer levels are predictive of adverse outcomes, including the development of acute respiratory distress syndrome (ARDS) and mortality [25]. D-dimer is therefore regarded as a crucial biomarker for identifying patients at higher risk of complications. IL-6 is a pro-inflammatory cytokine that plays a pivotal role in the immune response. In COVID-19, elevated IL-6 levels are correlated with the severity of the cytokine storm. Assessing these biomarkers provides insight into the extent of inflammation, tissue damage, and clotting abnormalities. Given the significance of these biomarkers in the clinical management of COVID-19, this study aimed to evaluate the effects of *B. coagulans* Unique IS-2 and *B. clausii* UBBC-07 on the levels of CRP, serum ferritin, LDH, D-dimer, and IL-6 in COVID-19 patients and on recovery.

## Materials and methods

Study design

This double-blind, randomized, placebo-controlled, parallel-group, three-arm study was conducted at Employees' State Insurance Corporation (ESIC) Hospital, India from March to August 2021. The study was conducted as per the guidelines of the International Conference on Harmonization-Good Clinical Practice (ICH-GCP), the Indian Council of Medical Research (ICMR) guidelines, and the principles of the Declaration of Helsinki. Approval for the study was obtained from the Institutional Ethics Committee of ESIC Medical College and Hospital (ESICMC/SNR/IEC-F0266/02/2021) on 02.02.2021 and the trial was registered prospectively with the Clinical Trials Registry - India (CTRI/2021/03/031720).

Study population

Sixty patients were enrolled in the study, of whom 20 patients were designated to receive *B. clausii* UBBC-07 suspension (2×109 spores/5 ml), 20 patients were designated to receive *B. coagulans* Unique IS-2 suspension (2×109 spores/5 mL), and 20 patients were designated to receive the placebo suspension. The patients had a mean age of 44 ± 14 years, with 38 (67.85%) being male and 16 (32.14%) female. All subjects in the study were of Indian origin.

Inclusion criteria

Patients aged over 18 years (age range: 18-80) of either gender, diagnosed with moderate COVID-19 symptoms confirmed through the reverse transcription-polymerase chain reaction (RT-PCR) method were included in the study. Moderate cases were defined as patients with pneumonia without signs of severe disease, characterized by symptoms such as dyspnea, hypoxia, fever, and cough, with oxygen saturation (SpO2) between 90% and ≤93% on room air, and a respiratory rate of ≥24 breaths per minute.

Exclusion criteria

Patients with a known history of hypersensitivity to probiotics, patients with HIV or hepatitis infection, and pregnant or lactating women were excluded from the study.

Randomization

After obtaining signed, written informed consent, eligible patients were admitted to the moderate COVID-19 ward and randomized equally into three groups of 20 patients each by computer-generated method. The groups were characteristically similar in terms of age and clinical demographics. All patients received standard COVID-19 treatment, which included remdesivir, steroids, anticoagulants, and supportive care following the Ministry of Health and Family Welfare (MOHFW), Government of India guidelines (version 5). In addition to the standard treatment, patients in the test groups received a 5 mL vial containing an oral suspension of either *Bacillus clausii* UBBC-07 or* Bacillus coagulans *Unique IS-2 (2 billion colony forming unit (CFU) spores) twice daily. Patients in the placebo group received identical-looking 5 mL vials of distilled water twice daily. The treatment duration was 14 days. The study medications were dispensed by a clinical pharmacologist, and all investigators were blinded to the treatment. Compliance was confirmed by counting empty vials at the end of the study.

Patients' condition at the time of admission

At the time of randomization, the predominant clinical manifestations included reduced oxygen saturation (SpO2 <94%) along with symptoms of dyspnea (shortness of breath), tachypnea (rapid breathing), fever, cough, and anosmia. Comorbidities were present in several patients: 12 were diabetic, 17 were hypertensive, and one had hypothyroidism. All patients received remdesivir, with six patients receiving intravenous infusions for 10 days while the remaining patients received the treatment for five days. The dosing regimen included a 200 mg loading dose on day one, followed by 100 mg for the subsequent four days.

Parameters assessed

The treatment period lasted for 14 days, during which the recovery was monitored by the treating physician. The biochemical parameters like CRP, LDH, and serum ferritin levels were assessed on day one, day seven, and day 14 of treatment. IL-6 and D-dimer levels were assessed at baseline and end of treatment on day 14.

Clinical parameters such as respiratory rate (RR) and SpO2 were recorded daily and compared on days one, three, seven, 11, and 14.

Adverse events potentially related to probiotic intake, such as gas, bloating, hypersensitivity reactions (e.g., rash, angioedema, and urticaria), were recorded. The progression from moderate to mild disease or advancement to severe forms during the treatment period was compared between the groups. Clinical improvements in COVID-19 symptoms and the incidence of adverse events, including bloating and hypersensitivity reactions, were monitored throughout the study.

Assessment of biochemical parameters

The serum was prepared by collecting 5 mL of blood in a plain vacutainer, allowing it to clot at room temperature for approximately 30 minutes, followed by centrifugation at 4000 rpm for five minutes. The serum was immediately processed for LDL and serum ferritin levels using the COBAS C311 Clinical Chemistry Analyzer (Roche Diagnostics, Rotkreuz, Switzerland). IL-6 levels were analyzed using Vitros 3600 (Ortho Clinical Diagnostics, Raritan, NJ). CRP was measured qualitatively through latex agglutination. Agglutination was considered as a positive test result indicating the presence of detectable levels of CRP in the serum whereas no agglutination was considered as a negative test result and indicated the absence of detectable levels of CRP. D-dimer levels were measured by collecting 2 mL of whole blood into a sodium citrate-containing vacutainer. The sample was processed on the same day using the Sysmex CS 1600 analyzer (Sysmex, Hyogo, Japan).

Statistical analysis

Demographic data and clinical characteristics were reported as mean ± standard deviation. Within-group analyses were performed using the Wilcoxon signed-rank test, while between-group comparisons were made using the Kruskal-Wallis test. A statistical power of 80% and a p-value of <0.05 was considered statistically significant. Data analysis was conducted using SPSS version 20 software (IBM Corp., Armonk, NY). The distribution of study variables, including laboratory biomarkers (CRP, LDH, IL-6, serum ferritin, and D-dimer), was evaluated using the Shapiro-Wilk test, which confirmed a non-normal distribution. Consequently, these biomarkers are presented as medians and ranges, and between-group comparisons were conducted using the Kruskal-Wallis test to account for the non-normality of the data. The sample size was determined to be 60, based on the number of patients admitted with moderate COVID-19 symptoms in the ward of the Department of General Medicine, ESIC Medical College and Hospital, Hyderabad.

## Results

**Figure 1:**
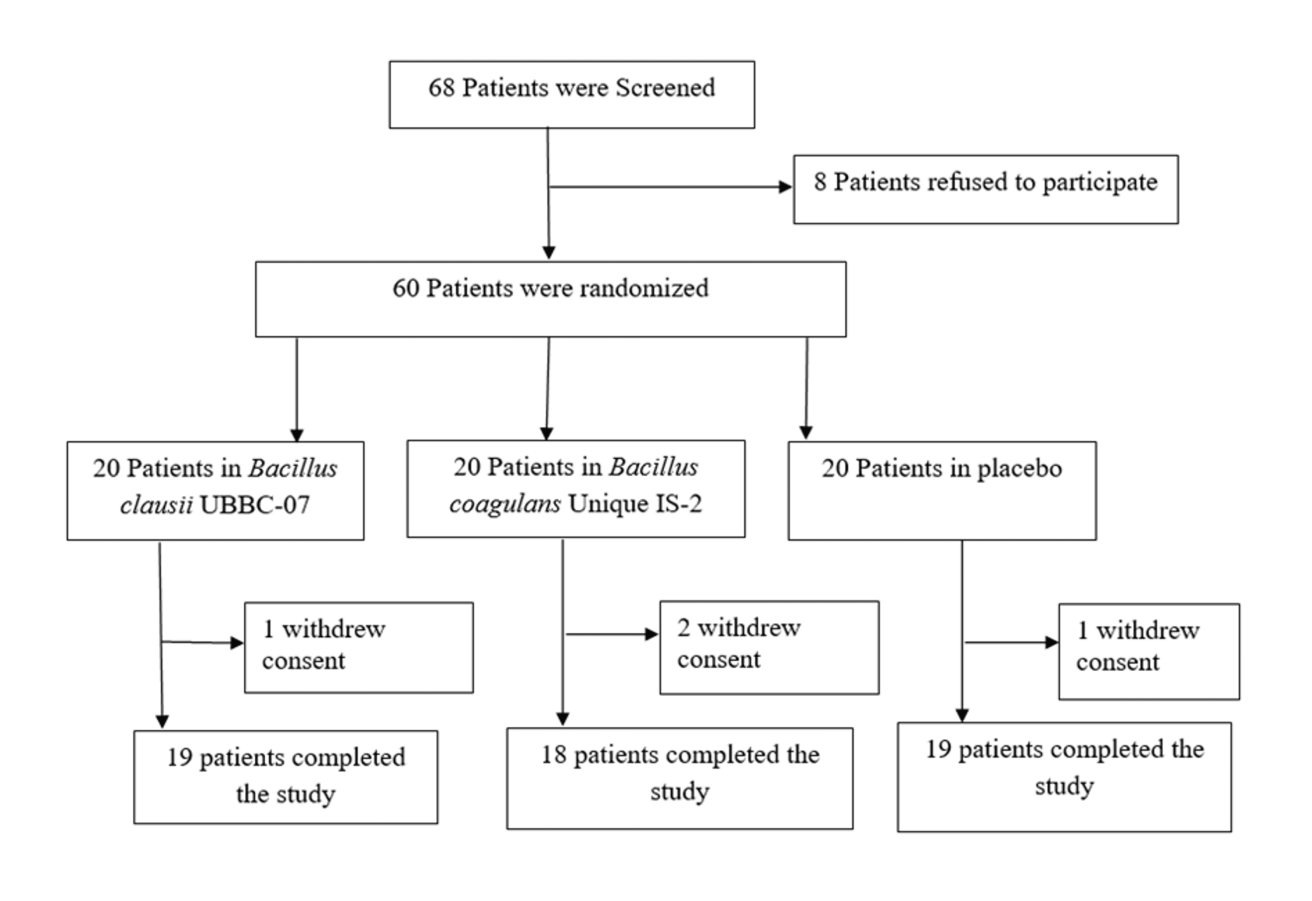
Study flowchart.

A total of 56 patients completed the study according to the protocol (per protocol population) (Table 1). Recovery and clinical outcomes from moderate COVID-19 infection were evaluated on days one, three, seven, 11, and 14 in each study group (Figure 1).

**Table 1:** Demographics depicting age and gender.

	Total patients (n = 56)	*B. clausii* UBBC-07 (n = 19)	*B. coagulans* Unique IS-2 (n = 18)	Placebo (n = 19)
Gender distribution (%)				
Male	38 (67.85)	13 (68.42)	12 (66.66)	13 (68.42)
Female	6 (32.14)	6 (31.57)	6 (33.33)	6 (31.57)
Age (years), mean ± SD				
21-80	44.2 ± 13.59	42.47 ± 11.95	45.78 ± 13.96	44.68 ± 15.23

Clinical assessments compared respiratory parameters such as RR and SpO2 across the groups. Additionally, symptoms like fever and cough were regularly monitored throughout the study. No statistically significant differences (p > 0.05) were observed between the treatment groups on the specified days. In the group treated with *Bacillus clausii* UBBC-07, 13 out of 19 patients recovered from moderate disease, with only two progressing to severe disease by day 14. Similarly, in the *Bacillus coagulans* Unique IS-2 group, 14 out of 18 patients recovered, while three advanced to the severe stage by day 14. In the placebo group, 14 out of 19 patients recovered from moderate disease, and two progressed to severe stage by day 14. Statistical significance is generally considered at a p-value less than 0.05. All groups demonstrated significant improvement (p < 0.05) in clinical parameters, particularly in SpO2 and RR. However, no significant differences (p > 0.05) were observed between the treatment groups on any of the specified days (Table 2).

**Table 2:** Assessment of oxygen saturation (SpO2) and respiratory rate on probiotic and placebo treatment.

Group	Respiratory parameters	Day 1	Day 3	Day 7	Day 11	Day 14	p-value
*B. clausii* UBBC-07 (n = 19)	SpO2	92±1	93±2	94±3	94±4	95±4	<0.05
	Respiratory rate	22±1	19±2	17±3	16±4	15±4	<0.05
*B. coagulans* Unique IS- 2 (n = 18)	SpO2	92±1	93±2	93±3	94±4	95±4	<0.05
	Respiratory rate	22±1	18±2	18±3	16±4	15±4	<0.05
Placebo (n = 19)	SpO2	93±1	93±2	94±3	95±3	95±3	<0.05
	Respiratory rate	21±2	19±2	17±4	16±4	16±4	<0.05
P-value		>0.05	>0.05	>0.05	>0.05	>0.05	

Since the data did not follow a normal distribution, the results are presented as medians along with their respective ranges. No significant differences were observed in other biomarkers, including LDH, IL-6, and CRP among the three groups on both days seven and 14 (Tables 3-5).

**Table 3:** Assessment of lactate dehydrogenase.

Group	Lactate dehydrogenase (U/L), range			
	Day 1	Day 7	Day 14	P-value (within-group)
*B. clausii* UBBC-07 (A) (n = 19)	409 (279-513)	220 (160-330)	200 (168-260)	Day 1-7 <0.01
				Day 1-14 <0.01
				Day 7-14 <0.01
*B. coagulans* Unique Is-2 (B) (n = 18)	394 (303-512)	270 (160-390)	200 (160-300)	Day 1-7 <0.01
				Day 1-14 <0.01
				Day 7-14 <0.01
Placebo (C) (n = 19)	412 (306-609)	240 (168-380)	200 (160-280)	Day 1-7 <0.01
				Day 1-14 <0.01
				Day 7-14 <0.01
P-value (between-group) - A vs. C	-	>0.05	>0.05	-
P-value (between-group) - B vs. C	-	>0.05	>0.05	
P-value (between-group) - A vs. B	-	>0.05	>0.05	

**Table 4:** Assessment of interleukin-6.

Groups	Interleukin-6 (pg/mL), median (range)		
	Day 1	Day 14	P-value (within-group)
*B. clausii* UBBC-07 (A) (n = 19)	3.67 (1.66-8.93)	4.95 (2.05-15.89)	Day 1-14 <0.03
*B. coagulans* Unique IS-2 (B) (n = 18)	1.82 (1.39-3.89)	4.40 (1.89-10.85)	Day 1-14 <0.02
Placebo (C) (n = 19)	1.99 (1.36-8.68)	3.69 (2.33-12.83)	Day 1-14 <0.04
P-value (between-group) - A vs. C	-	>0.05	-
P-value (between-group) - B vs. C	-	>0.05	
P-value (between-group) - A vs. B	-	>0.05	

**Table 5:** Qualitative assessment of CRP in the patient population (denoted as CRP positive & CRP negative).

	*B. clausii* UBBC-07, day 1	*B. clausii* UBBC-07, day 7	*B. clausii* UBBC-07, day 14
CRP positive	8	4	4
CRP negative	11	15	15
	*B. coagulans* Unique IS-2, day 1	*B. coagulans* Unique IS-2, day 7	*B. coagulans* Unique IS-2, day 14
CRP positive	10	6	5
CRP negative	8	12	13
	Placebo, day 1	Placebo, day 7	Placebo, day 14
CRP positive	10	9	4
CRP negative	9	10	15

Significant differences in serum ferritin levels were observed between *Bacillus clausii* UBBC-07 and placebo, as well as *Bacillus coagulans *Unique IS-2 and placebo, on days seven and 14. On day seven, the serum ferritin levels were reduced to 39.2% (345 μg/L) in the *Bacillus clausii* UBBC-07 group, 40.7% (350 μg/L) in the *Bacillus coagulans *Unique IS-2 group, and 30.9% (270 μg/L) in the placebo group. By day 14, serum ferritin levels were reduced to 75.6% (665 μg/L) in the *Bacillus clausii* UBBC-07 group, 73.3% (630 μg/L) in the *Bacillus coagulans* Unique IS-2 group, and 69.1% (595 μg/L) in the placebo group (Table 6).

**Table 6:** Assessment of serum ferritin levels.

Group	Serum ferritin (ng/L), median (range)			
	Day 1	Day 7	Day 14	P-value (within-group)
*B. clausii* UBBC-07 (A) (n = 19)	880 (396-1280)	535 (290-1030)	215 (160-850)	Day 1-7 <0.01
				Day 1-14 <0.01
				Day 7-14 <0.01
*B. coagulans* Unique IS-2 (B) (n = 18)	860 (390-1240)	510 (290-1030)	230 (140-890)	Day 1-7 <0.01
				Day 1-14 <0.01
				Day 7-14 <0.01
Placebo (C) (n = 19)	875 (400-1190)	605 (320-1320)	270 (190-940)	Day 1-7 <0.01
				Day 1-14 <0.01
				Day 7-14 <0.01
P-value (between-group) - A vs. C	-	<0.01	<0.01	-
P-value (between-group) - B vs. C	-	<0.01	<0.01	
P-value (between-group) - A vs. B	-	<0.08	<0.07	

D-dimer levels were significantly reduced by day 14 in the *Bacillus coagulans*Unique IS-2 group compared to the placebo group. Median reductions were 60.0% (0.36 μg/mL) in the *Bacillus coagulans* Unique IS-2 group, 36.8% (0.21 μg/mL) in the *Bacillus clausii* UBBC-07 group, and 34.4% (0.22 μg/mL) in the placebo group (Table 7).

**Table 7:** Assessment of D-dimer levels.

Groups	D-dimer (µg/L), median (range)		
	Day 1	Day 14	P-value (within-group)
*B. clausii* UBBC-07 (A) (n = 19)	0.57 (0.28-1042)	0.36 (0.15-1.12)	Day 1-14 <0.03
*B. coagulans* Unique IS-2 (B) (n = 18)	0.60 (0.38-1.08)	0.24 (0.10-0.78)	Day 1-14 <0.02
Placebo (C) (n = 19)	0.64 (0.39-1.39)	0.42 (0.13-1.12)	Day 1-14 <0.04
P-value (between-group) - A vs. C	-	<0.08	-
P-value (between-group) - B vs. C	-	<0.04	
P-value (between-group) - A vs. B	-	<0.07	

## Discussion

This study investigated the role of *Bacillus clausii* UBBC-07 and *Bacillus coagulans* Unique IS-2 in preventing and mitigating COVID-19 complications. Recovery rates from COVID-19 and the progression to severe disease were similar across all three groups, i.e., the two probiotic groups and the placebo group. Specifically, two out of 19 patients in the *B. clausii *UBBC-07 group, two out of 18 patients in the* B coagulans* Unique IS-2 group, and three out of 19 patients in the placebo group progressed to severe disease. Improvements in respiratory parameters, such as SpO2 and RR were also comparable across the groups. This similarity in recovery pattern is attributed to the standard treatment administered to all patients. Both the *B. coagulans* Unique IS-2 and *B. clausii* UBBC-07 groups demonstrated significant improvements, with marked reductions in ferritin and D-dimer levels compared to the placebo group. Elevated levels of D-dimer and ferritin are well-known to correlate with the need for mechanical ventilation and higher comorbidity rates. The observed reductions indicate that these probiotic strains may play a critical role in restoring balance to these key biomarkers. Elevated ferritin levels often signify an overactive immune response, commonly associated with a cytokine storm that triggers widespread inflammation, tissue damage, and a self-perpetuating pathogenic cycle. As a marker of iron metabolism and inflammation, ferritin reflects a hyperinflammatory state. In critically ill COVID-19 patients, elevated ferritin levels may lead to increased intracellular iron, fostering iron-dependent lipid peroxidation and causing cellular apoptosis through a process known as ferroptosis [26,27]. Similarly, D-dimer has emerged as a vital prognostic marker in COVID-19, with elevated levels linked to worse outcomes. Studies show that a D-dimer level above 1 µg/mL on admission increases mortality risk by 18-fold [28]. Elevated D-dimer levels have also been strongly associated with higher mortality in patients with infections or sepsis, as demonstrated in studies connecting increased D-dimer levels to 28-day mortality. Notably, significantly higher D-dimer levels have been observed in COVID-19 fatalities [29].

In the probiotic groups (*B. clausii *UBBC-07 and *B. coagulans* Unique IS-2), the reduction in D-dimer levels between day one and day 14 was statistically significant, with p-values of <0.03 and <0.02, respectively, highlighting the potential of these probiotics in mitigating inflammation and improving outcomes in COVID-19 patients by addressing key biomarkers associated with disease severity.

In comparison, although the placebo group also exhibited a reduction in D-dimer levels (p < 0.04), the decrease was less pronounced than in the probiotic groups. This indicates that the probiotics may have a more significant effect on reducing D-dimer levels than the placebo.

Similarly, while standard treatment alone resulted in a reduction in ferritin levels in the placebo group, both *B. coagulans* Unique IS-2 and *B. clausii *UBBC-07 demonstrated a more substantial improvement in balancing ferritin and D-dimer levels. These findings suggest that these probiotics may play a role in mitigating lung injury and supporting overall recovery more effectively than standard treatment alone.

CRP levels, indicative of active inflammation, showed no significant differences between the probiotic groups and the placebo group. While baseline CRP levels were elevated across all groups and decreased during follow-up, this reduction was primarily attributed to standard treatment. Furthermore, LDH and IL-6 levels remained unaffected by probiotic treatment, likely due to the specific pathways targeted by the probiotics. These results highlight the need for further research to elucidate the underlying mechanisms and their potential clinical implications. Reduced gut microbiota richness and diversity can result in immune dysregulation and intestinal inflammation, and exacerbate lung inflammation, potentially delaying SARS-CoV-2 clearance. It is suggested that probiotics may help support immune homeostasis by leveraging the bidirectional communication of the gut-lung axis [30]. Since the treatment duration was limited to two weeks, it is hypothesized that extended supplementation with* B. coagulans* Unique IS-2 and *B. clausii* UBBC-07 post-COVID-19 could further reduce inflammation, modulate the gut microbiota, and help restore normal levels of other biomarkers. One limitation of this study was that CRP was measured using a qualitative method rather than a quantitative one, due to the high patient load in the COVID-19 ward, which made it challenging to manage.

## Conclusions

Our findings suggest that *Bacillus clausii *UBBC-07 and *Bacillus coagulans* Unique IS-2 may serve as effective complementary therapies to mitigate tissue damage and thrombotic events in COVID-19 infections, with no adverse effects observed. We recommend further research on the prolonged supplementation of* B. coagulans* Unique IS-2 and *B. clausii* UBBC-07 post-COVID-19, as it may aid in reducing inflammation, modulating gut microbiota, and restoring normal biomarker levels. Additionally, extended supplementation could be evaluated for its potential to prevent recurrence and provide long-term support for immune system health.
